# Oxidative Stress in Polycystic Ovary Syndrome: Impact of Combined Oral Contraceptives

**DOI:** 10.3390/antiox13101168

**Published:** 2024-09-26

**Authors:** Nicolás Santander, Esteban G. Figueroa, Alejandro González-Candia, Manuel Maliqueo, Bárbara Echiburú, Nicolás Crisosto, Francisca Salas-Pérez

**Affiliations:** 1 Health Sciences Institute, Universidad de O’Higgins, Rancagua 282000, Chile; nicolas.santander@uoh.cl (N.S.); s.teban.fb@gmail.com (E.G.F.); alejandro.gonzalez@uoh.cl (A.G.-C.); 2 Laboratory of Endocrinology and Metabolism, Department of Internal Medicine, West Division, Faculty of Medicine, Universidad de Chile, Santiago 8350499, Chile; mmaliqueo@uchile.cl (M.M.); bechiburu@uchile.cl (B.E.); nicolascrisostok@gmail.com (N.C.); 3 Endocrinology Endocrinology Unit, Department of Medicine, Clínica Alemana de Santiago, Faculty of Medicine, Universidad del Desarrollo, Santiago 7650568, Chile

**Keywords:** metabolism, contraception, hormones, cardiovascular risk

## Abstract

Polycystic Ovary Syndrome (PCOS) is a complex hormonal disorder that is associated with heightened metabolic risks. While oxidative stress (OS) is known to play a role in PCOS, the precise nature of the relationship between PCOS and increased OS remains not entirely understood. Combined oral contraceptives (COCs) are the first-line treatment to regulate menstrual cycles and androgen levels, but their impact on oxidative stress requires further study. We conducted a transcriptomic analysis using RNAseq and assessed the levels of various oxidative stress (OS) markers in serum samples from women with PCOS and controls and whether they were using combined oral contraceptives (COCs), including enzymatic activities, FRAP, and 8-isoprostane (8-iso). A total of 359 genes were differentially expressed in women with PCOS compared to control women. Genes differentially expressed were enriched in functions related to inflammation and, interestingly, oxidative stress response. In controls, 8-iso levels were increased in women using COCs, whereas in women with PCOS, 8-iso levels were reduced in those using oral contraceptives (191.1 ± 97 vs. 26.4 ± 21 pg/mL, *p*: <0.0001). Correlation analyses showed a trend for a negative correlation between 8-iso and Ferriman score in women with PCOS consuming COCs (r = −0.86, *p* = 0.06) and a negative correlation between GSH and hyperandrogenism in women with PCOS (r = −0.89, *p* = 0.01). These results reveal the presence of lipid peroxidation in women with PCOS, which was modified by the use of COCs, providing new insights into the pathophysiology of PCOS in the Chilean population.

## 1. Introduction

Polycystic Ovary Syndrome (PCOS) is a complex endocrine disorder affecting 5–18% of women of reproductive age, with very heterogenous clinical manifestations including reproductive, metabolic, and psychological features [1,2]. The diagnosis is based on the presence of two of three criteria: clinical or biochemical hyperandrogenism, oligoovulation, and polycystic ovarian morphology [3]. Women with PCOS exhibit an increased risk of type 2 diabetes, non-alcoholic fatty liver disease, and cardiovascular disease [4,5]. This condition has a complex etiology and has been associated with a genetic and epigenetic predisposition, increased androgen exposure, neuroendocrine disturbances, ovarian dysfunction, obesity, dietary patterns, and oxidative stress [1,6]. However, the exact cause of PCOS is not fully understood. Still, it is believed to involve a combination of genetic and environmental factors, which may lead to an imbalance in redox homeostasis.

Oxidative stress (OS) is an imbalance between oxidants and antioxidant molecules. One of the primary cellular oxidant sources is that derived from oxygen called ROS, which are highly unstable and reactive molecules that are stabilized by gaining electrons. Physiologically, it maintains homeostasis in various cellular processes, including proliferation, differentiation, development, migration, and metabolism. However, excess ROS in pathological states reacts with lipids, proteins, and nucleic acids, leading to cellular dysfunction and OS apoptosis [7]. In this regard, the control of ROS is carried out by antioxidant machinery, which can be classified into enzymatic and non-enzymatic antioxidants. Enzymatic antioxidants include superoxide dismutase (SOD), catalase (CAT), and glutathione peroxidase (GPx), while non-enzymatic antioxidants include glutathione, thioredoxin, and various vitamins (vitamin C, A, and E) [8,9]. The relationship between PCOS and increased OS is not fully understood, but several contributing factors, such as insulin resistance or hyperandrogenemia, are thought to be involved. In PCOS patients, in addition, a decrease in serum total antioxidant capacity (TAC), which indicates the ability of antioxidants to counteract oxidative-stress-induced damage to cells, has been observed in individuals with PCOS, while total ROS were increased, indicating OS [10,11].

Treatment for PCOS comprises a wide range of interventions, such as lifestyle modifications and a pharmacological approach, according to the patient’s needs. In this context, insulin sensitizers such as metformin are recommended to improve metabolic disturbances. Combined oral contraceptives (COCs) are prescribed to normalize menstrual cycles and regulate androgen excess and its manifestations, including hirsutism and acne. COCs are a combination of low-dose estrogens and progestin, where using the lowest effective dose is advised to lower metabolic risks and adverse effects [12]. Ethinylestradiol (EE), a modified synthetic form of estradiol, is the most used hormone, which increases the synthesis of liver proteins, including lipoproteins, angiotensinogen, and estrogen-dependent clotting factors, which are associated with the main adverse events attributed to COCs [13]. On the other hand, progestins have been developed to reduce androgenic side-effects such as dienogest, a derivative of testosterone, which has anti-androgenic activity and fewer metabolic adverse effects [14]. International guidelines do not suggest one preparation over another, given that the current evidence is not sufficient to reveal a difference in efficacy between preparations [15].

The antioxidant effects of combined oral contraceptives (COCs) are still under investigation. However, it has been reported that COCs exhibited increased OS markers and changes in serum metabolome and high-sensitivity C-reactive protein, a marker of chronic inflammation, as well as MDA and lipid hydroperoxides [16,17,18]. Given that OS is associated with metabolic disturbances in PCOS, it has been hypothesized that OS may influence cardiometabolic risk [19], which in turn could be modulated by COC consumption. This work aimed to measure the antioxidant capacity and OS markers in women with PCOS treated with COCs.

## 2. Materials and Methods

### 2.1. Participants and Study Design

A total of 28 newly diagnosed Chilean women with PCOS and normoandrogenic controls were recruited at the Unit of Endocrinology and Reproductive Medicine, University of Chile, Santiago, Chile. Women with PCOS and controls were divided into two subgroups: users and non-users of combined oral contraceptives (COCs: dienogest 2 mg and ethinylestradiol 0.03 mg). The current study included women between 18 and 34 years old, with a body mass index (BMI) ranging from 18 kg/m^2^ to 35 kg/m^2^. Anthropometric measurements were recorded, blood samples were collected, and transvaginal ultrasound was performed to measure ovarian volume and follicle count. This study was conducted during the early follicular phase (between the third and seventh day of the menstrual cycle), except for women with amenorrhea who had blood sampling at any time. Clinical hyperandrogenism was determined using the Ferriman–Gallwey score, a clinical assessment tool for hair growth in androgen-dependent sites such as the lip, chin, chest, and others [20].

### 2.2. Inclusion and Exclusion Criteria

Inclusion criteria for women with PCOS were the following: (a) clinical (Ferriman–Gallwey score > 7.0), (b) serum testosterone > 0.8 ng/mL, or androstenedione > 2.46 ng/mL or free androgen index (FAI) > 5.0. In addition to hyperandrogenism, women with PCOS must present oligoovulation or polycystic ovarian morphology on ultrasound, with the presence of a follicle count per ovary of ≥20 or an ovarian volume ≥ 10 mL on either ovary, ensuring that no corpora lutea, cysts, or dominant follicles were present. Inclusion criteria for control women were the absence of hyperandrogenism and a history of regular menstruation. Exclusion criteria were pregnancy, lactation, vegetarianism, and veganism. Control and PCOS women with hematologic, infectious, or inflammatory diseases, ischemic coronary disease, diabetes, hypothyroidism, Cushing’s syndrome, use of antibiotics in the past six months, use of metformin, methotrexate, sulfasalazine, and medication for epilepsy or hypertension were excluded from the study. In addition, participants who consumed vitamin supplements were excluded due to their potential impact on oxidative stress markers.

### 2.3. Biochemical Analysis

After an overnight fast, venous blood samples were collected for subsequent laboratory procedures. Biochemical and lipid profiles, including total cholesterol, high-density lipoprotein cholesterol, and triglycerides, were determined by chemiluminescence (Ortho Clinical Diagnostic, Vitros 4600, Raritan, NJ, USA). Low-density lipoprotein cholesterol was calculated using the Friedewald formula [21]. Serum hormones such as testosterone, androstenedione, estradiol, and 17 hydroxyprogesterone (17-OHP) were analyzed by radioimmunoassay (Gamma counter, Berthold, Germany). Sex-hormone-binding globulin (SHBG), follicle-stimulating hormone (FSH), and luteinizing hormone (LH) were measured by immunoradiometric assay (IRMA) (Gamma counter, Berthold, Germany for SHBG and Gamma counter, Packard, TX, USA for LH and FSH). Anti-Müllerian hormone (AMH) was analyzed by ELISA (Euroimmun, Hamburg, Germany). Free androgen index (FAI) was calculated using the equation FAI (%) = (total testosterone/SHBG) × 100 [22]. Insulin resistance was assessed using the Homeostatic Model Assessment—Insulin Resistance (HOMA-IR) index: fasting insulin (mU/L) × plasma glucose (mmol/L)/22.5. Insulin was measured by chemiluminescence (Centauro XPT, Siemens, Munich, Germany). Plasma samples showing hemolysis were not considered for the analyses.

### 2.4. PBMC Isolation

Peripheral blood mononuclear cells (PBMCs) were isolated from whole blood previously collected in EDTA tubes, employing a density gradient centrifugation over Histopaque^®^-1077 (Sigma-Aldrich, St. Louis, MO, USA) according to manufacturer’s instructions. Subsequently, PBMCs were transferred to a labeled tube containing 100 µL of RNAlater^®^ (Sigma-Aldrich, St. Louis, MO, USA) and stored at −80 °C until RNA isolation.

### 2.5. RNA Sequencing

We used a subset of the full cohort for RNA sequencing due to costs. This subset is representative of the whole cohort in terms of inclusion and exclusion criteria, and characteristics are shown. Total RNA from PBMCs was isolated and extracted with PureLink™ RNA Micro Kit (ThermoFisher Scientific, Waltham, MA, USA) following the manufacturer’s protocol from animal cells. Sequencing libraries were constructed from total RNA with RIN > 8 using the NEBNext Ultra II RNA Library Prep Kit (New England Biolabs, Ipswich, MA, USA). Unstranded libraries were sequenced using a HiSeq4000 system (Illumina, San Diego, CA, USA) to produce ~40 million 150 pb paired-end reads. After demultiplexing, fastq files were aligned to the human genome (GRCh38) with Rsubread 2.10 and quantified using FeatureCounts. Differential expression analysis was performed with DESeq2 1.36. Volcano plots were rendered using the Enhanced Volcano 1.14 package, and heat maps were made using the pheatmap 1.0 package. Overrepresentation analysis was performed using PANTHER 17.0, while gene set enrichment analyses were performed with GSEA 4.2. Data has been deposited in GEO under accession GSE262735.

### 2.6. Metabolite Measurement

The total glutathione concentration was determined with the GSH assay kit (ab239709, Abcam, Cambridge, UK) and the 8-isoprostane concentration was measured by ELISA (516351, Cayman Chemical Company, Ann Arbor, MI, USA) following the manufacturers’ guidelines.

### 2.7. Ferric Reducing Ability of Plasma (FRAP)

The total antioxidant capacity of plasma was assessed by the ferric reducing ability of plasma. FRAP solution was prepared by mixing 300 mM of acetate buffer (pH 3.6), 10 mM of 2,4,6-tri(2-pyridyl)-1,3,5-triazine (TPTZ) solution, and 20 mM of FeCl_3_·6H_2_O in a 10:1:1 ratio and subsequently heating the resultant mixture to 37 °C. The reaction mixture was composed of 750 µL FRAP solution, 75 µL H_2_O, and 25 µL of the sample and then incubated at 25 °C in darkness for 30 min, and the absorbance was read at 593 nm. Results were interpolated into a standard curve of FeSO_4_.

### 2.8. Enzymatic Activity

Enzymatic activities in plasma were measured using specific assays with the following kits, according to the manufacturer’s instructions: glutathione peroxidase assay kit (703102, Cayman Chemical Company, Ann Arbor, MI, USA), catalase activity assay kit (707002, Cayman Chemical Company, Ann Arbor, MI, USA), and superoxide dismutase activity assay kit (706002, Cayman Chemical Company, Ann Arbor, MI, USA).

### 2.9. Statistics

The normal distribution of quantitative variables was tested using Kolmogorov–Smirnov and Shapiro–Wilk tests. Clinical and laboratory characteristics were compared between cases and controls using unpaired Student’s *t*-test for normally distributed data or Mann–Whitney U test for data with a non-normal distribution. One-way ANOVA was used to analyze three or more independent groups, followed by Tukey’s post hoc test for multiple comparisons. Descriptive statistics are presented as mean ± standard deviation (SD) or median (25th–75th percentile values). Categorical variables are expressed as n (%). Sequencing data was analyzed by fitting a generalized linear model assuming a negative binomial distribution and applying a Wald test. We considered genes to be differentially expressed at an FDR < 0.1.

Continuous variables were compared using two-way ANOVA to test the effect of the condition (control/PCOS) and oral contraceptive usage (none/contra). We report on the effects of each of those factors and their interaction. We considered the standard *p* < 0.05 statistically significant in these analyses. Correlations between continuous variables were quantified using Pearson’s coefficient. We report *p*-values for correlation coefficients ≥ 0.7. All statistical calculations were performed in the R statistical environment (v. 4.2.2).

## 3. Results

### 3.1. Anthropometric, Clinical, and Biochemical Data of the Sample

A total of 18 women were included in the study, with 9 newly diagnosed with PCOS and 9 serving as controls. The anthropometric and clinical data of the categorized groups are summarized in Table 1, with the Ferriman indicator being significantly higher in the PCOS group (12 vs. 3) compared to the control group. There was no difference in age distribution between the groups (*p* = 0.883); however, significant differences were observed in BMI, which was notably higher in the PCOS group (30.5 ± 4 vs. 22.5 ± 2.2 kg/m^2^) compared to the control group (*p* = 0.008), along with a slight increase in systolic blood pressure (SBP 124.4 ± 4 vs. 105.5 ± 9 mmHg). No significant differences were observed in other clinical data (glucose, insulin, HOMA-IR index, total cholesterol, HDL, LDL, and triglycerides) between the PCOS and control groups.

Endocrine markers were measured in the study population (Table 2). Ferriman score was significantly increased in women with PCOS compared to controls. Total testosterone levels did not exhibit differences between groups. However, SHBG levels were significantly decreased, which, in turn, increased the free androgen index in the PCOS group. No differences were found between groups for androstenedione, FSH, LH, 17-OHP, and AMH.

### 3.2. Transcriptomic Changes in PCOS

PCOS is a complex condition involving hormonal disarrangements that promote the development of metabolic syndrome. We collected peripheral blood mononuclear cells for high-quality transcriptomic profiling using RNA sequencing to survey the molecular responses to these altered responses.

In this study, we recruited 18 newly diagnosed women with PCOS and controls, as previously described (Table 1 and Table 2).

**Figure 1:**
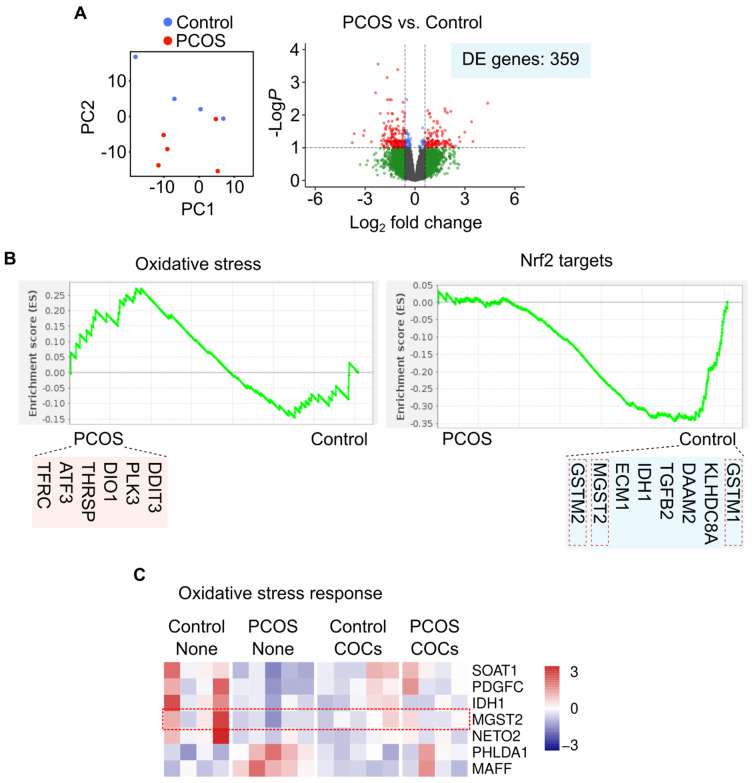
Transcriptomic profiling of PBMCs from women with PCOS and controls. (**A**) General differences between control and PCOS samples at the transcriptomic level are shown in PCA and Volcano plot. Statistical significance is determined by DESeq2. (**B**) Gene Set Expression Analysis reveals enrichment of terms associated with redox signaling in PCOS samples. (**C**) Heatmap showing PCOS-upregulated genes involved in the oxidative stress response. Color scale indicates z-score of expression levels.

Transcriptomic profiles of PBMCs from control women differed from those of women with PCOS, showing differential expression of 359 genes (Figure 1A). Genes differentially expressed were enriched in functions related to inflammation and, interestingly, oxidative stress response. The latter has been associated with PCOS and its metabolic phenotypes. Next, we used GSEA to analyze the association of PCOS with the expression of genes involved in the oxidative stress response beyond differential expression. We observed an association between PCOS samples and the expression of oxidative-stress-responsive genes, with a robust inverse association observed between the PCOS condition and genes regulated directly by Nrf2 (Figure 1B). Moreover, women with PCOS showed lower expression of several enzymes with glutathione transferase activity of the microsomal class (Figure 1B).

The use of oral contraceptives has been associated with changes in the oxidative status of plasma in women in different cohorts [23,24]; therefore, we subsequently analyzed women with or without PCOS who were using oral contraceptives (COCs) at the time of recruitment. We observed that several genes that respond to cellular oxidative stress and are direct targets of Nrf2 showed differential expression between controls and women with PCOS (Figure 1C). Notably, the use of oral contraceptives was associated with intermediate expression levels of these genes (Figure 1C).

These results suggest that PBMCs from women with metabolically compromised PCOS are exposed to an oxidative environment. Therefore, we evaluated these women’s redox state markers in plasma.

### 3.3. Antioxidant Enzymes

The oxidative status results from an intricate network of metabolic pathways in the cell and the extracellular space, controlled by the activity of several enzymes catalyzing redox reactions. We determined the main enzymatic activities responsible for the metabolism of critical reactive oxygen species in plasma from women with or without PCOS who used oral contraceptives. These measurements were performed in the whole cohort (28 samples), characterized in Tables S1 and S2. This extended cohort shows similar characteristics to the smaller cohort used for sequencing: PCOS women have higher BMI and SBP, while women using COCs have higher plasma cholesterol.

**Figure 2:**
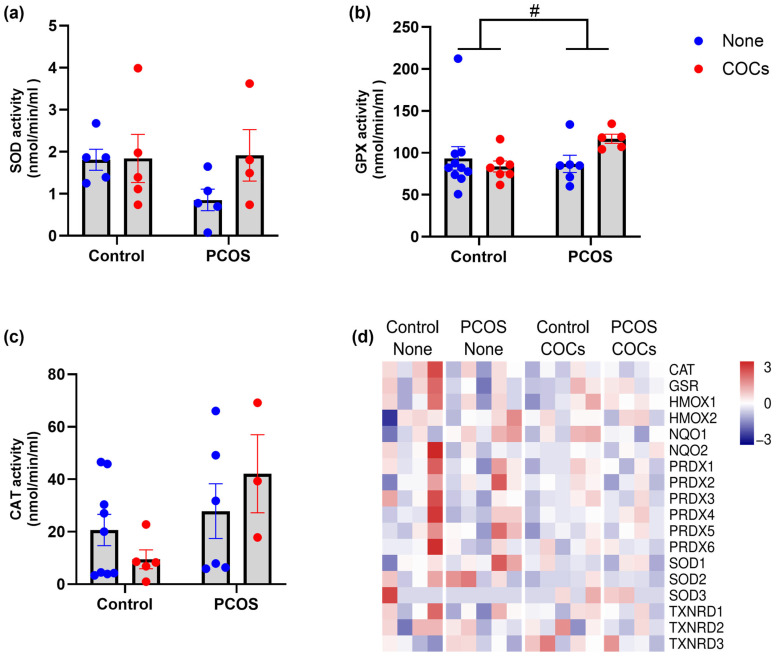
Antioxidant capacity in plasma samples from women with PCOS and controls, with or without COCs. (**a**) Superoxide dismutase (SOD) activity in plasma, (**b**) glutathione peroxidase (GPX) activity in plasma, and (**c**) catalase (CAT) activity in plasma. (**d**) Transcriptional profiling of antioxidant enzymes. Data expressed as mean ± S.E.M. and compared using a two-way ANOVA testing the effect of the condition (control/PCOS) and oral contraceptive usage (none/COCs). Significant interaction (*p* ≤ 0.05) control vs. PCOS. # *p* < 0.05.

Among antioxidant activities, we assessed the plasma enzymatic activities of superoxide dismutase (SOD), glutathione peroxidase (GPX), and catalase (CAT) (Figure 2a–c). No significant impact of the PCOS condition or the use of oral contraceptives was observed in any of the enzyme activities (Figure 2a–c).

To gain insight into other antioxidant enzymes, we explored the expression levels of enzymes involved in the principal reactions of the antioxidant system, including heme catabolism, quinone oxidation, and thioredoxin reduction. Antioxidant enzymes such as catalase (CAT), glutathione reductase (GSR), heme oxygenases 1 and 2 (HMOX1 and HMOX2), NAD(P)H quinone dehydrogenases 1 and 2 (NQO1 and NQO2), peroxiredoxins (PRDX1, PRDX2, PRDX3, PRDX4, PRDX5, and PRDX6), superoxide dismutases (SOD1, SOD2, and SOD3), and thioredoxin reductases (TXNRD1, TXNRD2, and TXNRD3) were evaluated. In line with the observations of enzymatic activity, the expression levels of all major antioxidant enzymes did not differ among any of the groups studied (Figure 2d).

### 3.4. Markers of the Oxidative Status in Plasma

**Figure 3:**
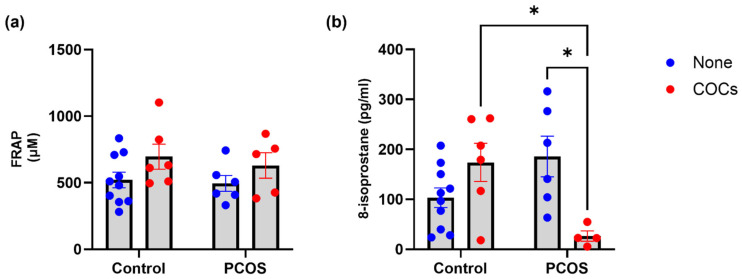
Stress oxidative markers. (**a**) Ferric reducing ability of plasma (FRAP) and (**b**) 8-isoprostane in woman plasma. Data expressed as mean ± S.E.M. and compared using a two-way ANOVA. Significant difference (*p* ≤ 0.05). * *p* < 0.05.

We measured total antioxidant capacity using the FRAP method, and no significant changes were observed among groups in samples of women using oral contraceptives (Figure 3a). In contrast, levels of 8-isoprostane, a derivative of arachidonic acid produced by peroxidation, showed distinct trends in plasma from women with PCOS and controls (Figure 3b). In control samples, levels of this peroxidation product were increased in women using oral contraceptives. In contrast, in women with PCOS, 8-isoprostane levels were reduced in those using oral contraceptives (191.1 ± 97 vs. 26.4 ± 21 pg/mL, *p*: <0.0001). This interaction suggests that oral contraceptives have different effects in women with PCOS than in controls on the production of lipid peroxides.

### 3.5. Glutathione Metabolism

Glutathione is one of the most important regulators of the cellular redox status, as it provides available -SH groups for oxidation at the mM range in the intracellular space. It is also found at relatively high concentrations in human plasma and might serve as a sink for reactive species. 

**Figure 4:**
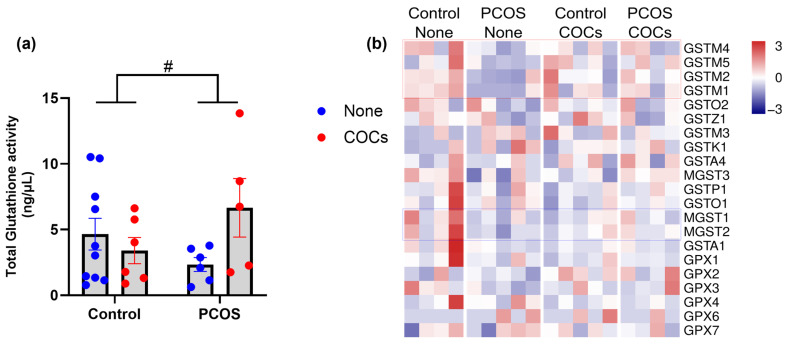
Glutathione metabolism. (**a**) Total glutathione in plasma. Data expressed as mean ± S.E.M. and compared using a two-way ANOVA. Significant differences (*p* ≤ 0.05). (**b**) Transcriptional profiling of glutathione metabolism enzymes. Differentially expressed enzymes in PCOS women are highlighted with dotted rectangles. Significant interaction (*p* ≤ 0.05) control vs. PCOS. # *p* < 0.05.

We measured glutathione levels in plasma from women in our cohort and observed that the PCOS condition was associated with a slight change in this parameter (Figure 4a). Use of oral contraceptives led to no changes in plasma glutathione in control women, but contraceptive use was associated with an increase in plasma glutathione in women with PCOS (Figure 4a; *p*-value for the interaction = 0.05). 

Next, we determined the expression levels of the enzymes involved in glutathione metabolism using our sequencing dataset (Figure 4b). Consistent with a minor change in plasma GPX activity, we did not observe changes in the expression of enzymes of the GPX family.

On the other hand, expression levels of most glutathione transferases of the Mu family (GSTM) showed down-regulation in samples from women with PCOS not using oral contraceptives when compared to control samples (Figure 4b, red dotted rectangle). Notably, these changes in expression were significantly attenuated in women with PCOS who used oral contraceptives. These enzymes conjugate glutathione with electrophilic compounds, including reactive species.

Finally, expression levels of two members of the microsomal family of glutathione transferases (MGST) were also reduced in women with PCOS without oral contraceptives (Figure 4b, blue dotted rectangle). These enzymes catalyze steps in synthesizing eicosanoids (leukotrienes and prostaglandins) and reducing lipid peroxides. Oral contraceptives did not normalize the expression of these enzymes in women with PCOS (Figure 4b, blue dotted rectangle).

### 3.6. Estimating the Effect of Androgens on Levels of Oxidative Status Markers

One of the main features of PCOS in our population is overexposure to androgens, and oral contraceptives reduce the Ferriman score independently of other clinical characteristics in women with PCOS (Table 1). Based on these characteristics of our cohort, we sought to study the contribution of androgens on the analyzed parameters, focusing on FRAP, 8-isoprostane, and glutathione-related markers.

**Figure 5:**
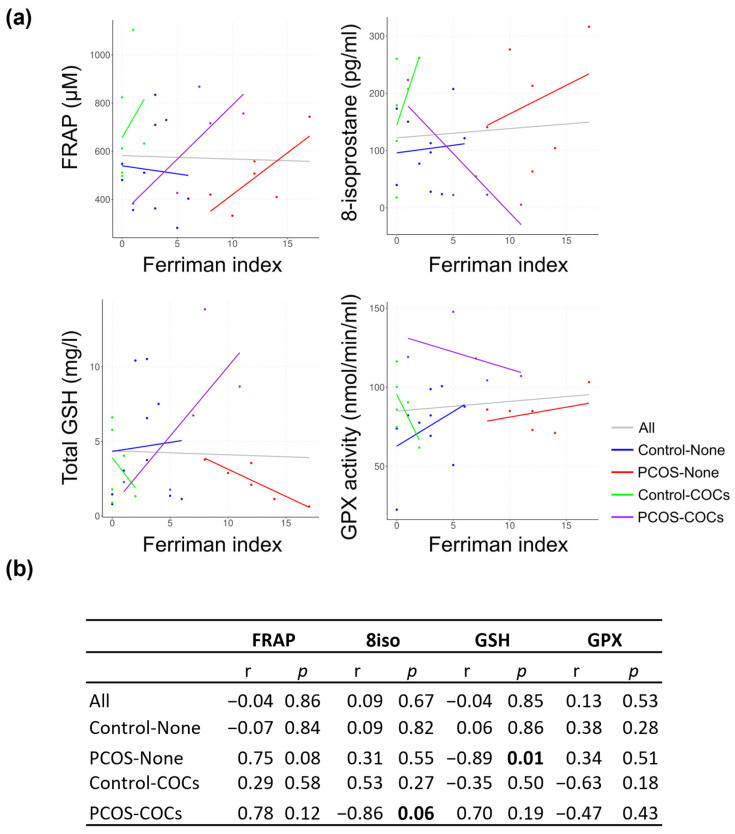
Correlation analysis between oxidative stress and hyperandrogenism (Ferriman index). (**a**) Correlation analysis between oxidative stress markers, antioxidant activity, Ferriman index, and (**b**) table of correlation coefficients and *p*-value of correlation. Abbreviations: FRAP: ferric reducing ability of plasma; Total GSH: total glutathione; GPX activity: glutathione peroxidase; r: Pearson’s correlation coefficient; *p*-values < 0.05 were considered statistically significant.

We determined the correlation of the Ferriman index with the parameters of interest in our cohort, either in all samples or in each of the groups separately (Figure 5). When considering all samples together, there was no correlation between the Ferriman index and any of the parameters evaluated (Figure 5, gray lines). Notably, by analyzing correlations separately in women with or without PCOS who used oral contraceptives, we observed significant correlations between the Ferriman score and some parameters related to oxidative stress. For FRAP, a positive trend in the correlation with the Ferriman index was observed in the PCOS group (red line) (r = 0.75, *p* = 0.08). Consistently with the results in Figure 3b, we observed opposite correlations in the levels of 8-isoprostane and in the Ferriman score in women with PCOS who did not use oral contraceptives (red lines; r = 0.31, *p* = 0.5) compared to those who used contraceptives (purple lines; r = −0.86, *p* = 0.06). Notably, an opposite correlation was also observed in these groups for total GSH and the Ferriman score: a negative correlation was observed in women with PCOS who did not use oral contraceptives (red line; r = −0.89, *p* = 0.01), whereas there was a positive correlation in women with PCOS who used oral contraceptives.

These results provide support for the effect of oral contraceptives on the levels of plasmatic markers of redox status, specifically in women with PCOS. This effect might be mediated by changes in the levels and the contribution of androgens to the PCOS phenotype.

## 4. Discussion

In this study, we demonstrated that COCs decreased oxidative stress (OS) (lipid peroxidation) and increased glutathione bioavailability at the plasma level, which is associated with reduced hyperandrogenism. This study provides new insights into the pathophysiology of PCOS in the Chilean population. PCOS is recognized as a chronic systemic, endocrine, and metabolic disorder, where the combined administration of dienogest (progestogen) and ethinylestradiol (estrogens) in our cohort decreased BMI to values close to our control group. In addition, this treatment increased HDL-cholesterol and total cholesterol levels. In addition, the combined oral therapy decreased insulin, reduced DBP, and lowered the Ferriman score associated with body areas sensitive to androgens in women diagnosed with PCOS. This syndrome is a common gynecological and endocrine disorder associated with overweight and insulin resistance that generates metabolic problems such as dyslipidemia and type 2 diabetes in the short term [25]. Significantly higher insulin rates correlate with the decline in ovarian function and hepatic SHBG levels by induction of Insulin-like growth factor 1 (IGF-1) [26], increasing androgen’s availability. However, as previously described, oral combination therapy increased SHBG levels, reducing free testosterone [27]. On the other hand, hyperandrogenism is the fundamental endocrine abnormality of PCOS and serves as the essential diagnostic criterion of the syndrome [28]. Our group of patients showed a reduction in androstenedione, free androgen index, and AMH, indicating the normalization of androgen metabolism with contraceptive therapy. Two mechanisms have been proposed to explain this effect. The first mechanism involves the impact of contraceptives on the inhibition of androgens of ovarian origin through the inhibition of folliculogenesis [29], either by suppressing the secretion of pituitary gonadotrophin [30] or by directly influencing ovarian follicle development with a weaker antigonadotrophic effect [31]. The second mechanism is based on regulating adrenal steroidogenesis [32]. Previous antecedents have shown that the use of oral contraceptives can lead to a decrease in the serum concentration of dehydroepiandrosterone sulfate (DHEAS), a precursor of different androgens, or an increase in the levels of androgen-binding proteins such as SHBG [33]. Finally, the effect of the COCs on folliculogenesis significantly decreases androgen production. This mechanism has been confirmed in both healthy women and PCOS patients.

The exact cause of PCOS is not fully understood, but it is believed to encompass a combination of genetic and environmental factors, such as cellular inflammation and OS [34]. We conducted a transcriptomic analysis on PBMCs to determine the peripheral redox status. Our study observed an association between PCOS samples and the expression of oxidative-stress-responsive genes, with a robust inverse association observed between the PCOS condition and genes regulated directly by nuclear factor-erythroid 2-related factor (Nrf2). This transcriptional factor is a key molecule because it regulates the cellular defense against oxidative insults through the expression of genes involved in OS response [35]. Under basal conditions, Nrf2 is sequestered by Keap1, but upon activation, Nrf2 dissociates from Keap1 and translocates to the nucleus, where it heterodimerizes with small Maf proteins. This complex binds to the antioxidant response element (ARE) to induce the expression of genes encoding enzymes essential for detoxifying ROS and other oxidants, thereby playing a critical role in maintaining redox homeostasis [36,37]. The Nrf2 pathway has previously been linked to PCOS in line with our findings. It has been reported to be decreased in both adipose tissue samples from women with PCOS and the letrozole-induced model of PCOS [38,39].

Additionally, our study found that PCOS patients showed a trend toward a decrease in total SOD levels compared to the control group, with no changes in the activity of CAT and GPx. Furthermore, no differences were observed in the administration of COCs in the variables analyzed. One explanation is methodological and is based on the presence of false positives resulting from hemolyzed plasma samples. This led to a differential number of samples, as observed in colorimetric methodologies, ultimately affecting the statistical robustness of the data. Secondly, while all the antioxidant enzymes analyzed have Nrf2 binding sites in their promoter regions, this is not sufficient because they also have differential transcriptional activators and respond differently to oxidative stress [40].

Androgen excess can promote a pro-inflammatory cellular environment and metabolic syndrome, increasing ROS production and oxidative stress [11]. Therefore, ROS can bind and oxidize different cellular compounds (e.g., lipid, DNA, protein, and cellular membrane, among others), thus modifying their structure and function [41]. Although no differences were found in the total antioxidant capacity in the plasma of women with PCOS, COC therapy decreased the concentration of 8-isoprostanes in plasma, one of the primary lipid mediators generated by ROS [42]. However, the effects of combined estrogen and progestin therapy on OS are controversial, with many studies showing opposite effects [43]. Estrogens display antioxidant activity by inhibiting the expression and function of the NADPH oxidase and inducing the activation of antioxidant enzymes such as those of the SOD complex [44]. On the other hand, the antioxidant effects of estrogens are counteracted by progestins, which activate prooxidant enzymes such as xanthine oxidase and inhibit the expression and activity of antioxidant enzymes such as extracellular superoxide dismutase (ecSOD) [45]. Moreover, it is essential to consider the inherent prooxidant effect of estrogens on the overall redox balance. Estrogens can act as prooxidant molecules due to their potential to be metabolized and autoxidized by cytochrome P450 enzymes, producing orthoquinone, unstable molecules capable of generating ROS [46]. Although the molecules used in this therapy have opposite anti/pro-oxidant effects, the final effect is dose-dependent [47] and is influenced by the intracellular redox balance. A reduced intracellular environment generates less reactive pro-oxidant sources, resulting in less oxidative stress.

Glutathione (GSH) is the most abundant antioxidant molecule and is crucial to maintaining redox potential in tissues, cells, and individual compartments [48]. COCs increased plasma total glutathione levels and glutathione S-transferase gene clusters compared to our cohort of PCOS women. The induction of this pathway can directly contribute to the plasma redox state through various mechanisms. The first is that the glutathione molecule can function as a scavenger of free radicals or electrophilic molecules, decreasing their bioavailability [49], and can directly contribute to providing electrons for enzymes such as glutathione peroxidase family proteins [50]. Finally, this study associated the increase in 8-isoprostanes with the decrease in the bioavailability of total plasma glutathione and the hyperandrogenism phenotype in our group of women with PCOS. In contrast, COC therapy increased total glutathione levels and decreased the plasma concentration of 8-isoprostanes, decreasing the plasma OS. While the association between OS and PCOS is increasingly recognized, further research is needed to fully elucidate the role of OS in the pathophysiology of PCOS. Investigating the function of cell organelles such as mitochondria, pro-oxidant sources, and endogenous antioxidants may provide further insights into the metabolic and hormonal dysregulation associated with this syndrome.

This study has some limitations. First, the study was conducted with a reduced sample size, and a higher BMI in the PCOS group could be a confounding factor. Higher BMI is a common feature in women with this syndrome; however, it is associated with metabolic and hormonal parameters, as well as inflammation markers. Therefore, these results should be analyzed in larger samples to validate the findings derived from RNA-seq analysis. In addition, other potential confounders include lifestyle factors, such as diet and physical activity, which could impact these analyses. Controlling for these confounders will be crucial in future studies to ensure more accurate and interpretable results. Despite these limitations, this study contributes to the understanding of contraceptives in PCOS and their potential impact on oxidative stress.

## 5. Conclusions

Polycystic Ovary Syndrome (PCOS) is a prevalent endocrine disorder in women of reproductive age, characterized by a range of clinical symptoms and phenotypes resulting from hormonal imbalances. In this study, we observed changes in glutathione metabolism and lipid peroxidation dependent on oral contraceptive consumption, providing new insights into the pathophysiology of PCOS in the Chilean population. These findings highlight the need for further research to elucidate the mechanisms by which oxidative stress influences PCOS. Future studies should explore the long-term effects of different contraceptive formulations on oxidative stress markers and metabolic health in women with PCOS. Additionally, these insights could lead to developing targeted antioxidant therapies as a complementary treatment strategy to improve clinical outcomes and enhance the quality of life in women with PCOS.

## Figures and Tables

**Table 1:** Anthropometric and clinical data of the study population at baseline.

Parameter	Control (*n* = 4)	Control—COCs (*n* = 5)	PCOS (*n* = 5)	PCOS—COCs (*n* = 4)	*p*-Value
Age (years)	25.8 ± 3.3	26.6 ± 4.8	25.0 ± 6.1	24.3 ± 2.8	ns
BMI (kg/m^2^)	22.5 ± 2.2	23.9 ± 3.7 *	30.5 ± 4 ^#^	25.8 ± 1.1	0.008
Ferriman–Gallwey score system	3 (2.25–3.75)	0 (0–0.15) *	12 (11–15.5) ^#^	7.5 (2.5–10.3) *	<0.001
SBP (mmHg)	105.5 ± 9	118.4 ± 10	124.4 ± 4 ^#^	117 ± 9	0.02
DBP (mmHg)	65.3 ± 6	73.6 ± 6	72.8 ± 7.3	72.8 ± 5.5	ns
Glucose (mg/dL)	82 ± 5.5	80.8 ± 4	85.4 ± 2.9	83.5 ± 6.6	ns
Insulin (mU/L)	7.5 ± 3.1	9.1 ± 2.6	14.6 ± 8.1	11.1 ± 6.8	ns
HOMA-IR index	1.55 ± 0.7	1.82 ± 0.5	3.06 ± 1.7	2.35 ± 1.6	ns
Total Chol (mg/dL)	149.8 ± 20	195 ± 50	163 ± 39	209.5 ± 34	0.06
HDL-Chol (mg/dL)	50.7 (41–65)	74.4 (61–103) *	43.4 (39–52)	63.4 (54–78)	0.01
LDL-Chol (mg/dL)	78.1 (57–109)	72.8 (60–128)	100 (64–120)	112 (97–142)	ns
Triglycerides (mg/dL)	81.2 ± 26	123.2 ± 47	122 ± 62	114.5 ± 65	ns

Variables are shown as mean ± SD or %. Variables with skewed distribution are shown as median (25th–75th percentile values). *p*-values were calculated using one-way ANOVA; *p* < 0.05 was considered statistically significant. For Tukey post hoc, ^#^ *p* < 0.05 compared to the control group and * *p* < 0.05 in contrast to the PCOS group. BMI: body mass index; SBP: systolic blood pressure; DBP: diastolic blood pressure; HOMA-IR index: Homeostatic Model Assessment—Insulin Resistance index. Ferriman–Gallwey score system: scale for grading hirsutism with a cut-off value of 6, indicating mild hirsutism; ns represents not significant.

**Table 2:** Hormonal parameters of the study population at baseline.

Parameter	Control (*n* = 4)	Control—COCs (*n* = 5)	PCOS (*n* = 5)	PCOS—COCs (*n* = 4)	*p*-Value
Testosterone (ng/mL)	0.5 ± 0.1	0.6 ± 0.1	1.1 ± 1	0.68 ± 0.3	ns
SHBG (nmol/L)	54.8 ± 15	146 ± 60 *	22.1 ± 9	120.4 ± 95	0.01
FAI (%)	3.5 ± 1.0	1.7 ± 0.9 *	20.5 ± 18.2	3.4 ± 2.6	0.03
Androstenedione (ng/mL)	3.2 ± 0.7	2.6 ± 0.3 *	4.5 ± 1.4	2.8 ± 0.7	0.02
FSH (mUI/mL)	5.4 ± 1.5	7.1 ± 2.2	6 ± 2.6	5.3 ± 2.5	ns
LH (mUI/mL)	5 ± 1.2	4.1 ± 2.9	12.1 ± 11.8	5.8 ± 4.3	ns
Estradiol (pg/mL)	57.6 ± 8.5	75.1 ± 23.4	132.9 ± 159.6	77.5 ± 54.1	ns
17-OHP (ng/mL)	1.6 ± 0.2	1.4 ± 0.3	2.3 ± 1.4	1.7 ± 0.3	ns
AMH (ng/mL)	4.84 ± 3.5	3.26 ± 0.9 *	7.61 ± 2.6	4.9 ± 1.2	0.05

Variables are shown as mean ± SD or %, as appropriate. *p*-values were calculated using one-way ANOVA; *p* < 0.05 was considered statistically significant. For Tukey post hoc, *p* < 0.05 compared to the control group and * *p* < 0.05 compared to the PCOS group. SHBG: sex-hormone-binding globulin; FAI: free androgen index; FSH: follicle-stimulating hormone; LH: luteinizing hormone; 17-OHP: 17 hydroxyprogesterone; AMH: anti-Müllerian hormone; ns represents not significant.

## Data Availability

Sequencing data generated in this study has been deposited in GEO Accession Number GSE262735.

## Supplementary Materials

The following supporting information can be downloaded at: https://www.mdpi.com/article/10.3390/antiox13101168/s1, Supplementary Table S1. Anthropometric and clinical data of the study population; Supplementary Table S2. Hormonal parameters of the study population.
