# Supplementary material for: Oxidative Stress in Polycystic Ovary Syndrome: Impact of Combined Oral Contraceptives
Source: Antioxidants (Basel). 2024 Sep 26;13(10):1168. doi: 10.3390/antiox13101168 (PMC11505064; doi:10.3390/antiox13101168)
Supplement: Supplementary file 1 [file antioxidants-13-01168-s001.zip › antioxidants-3180138-supplementary.pdf]

Supplementary Table S1. Anthropometric and clinical data of the study population.

Parameter	**Control** (n=10)	**Control-COCs** (n=6)	**PCOS** (n=7)	**PCOS-COCs** (n=5)	**p-Value**
Age (years)	26.1 ± 4.4	27.2 ± 4.5	24.7 ± 5.5	24.4 ± 2.4	0.620
BMI (kg/m^2)	23.6 ± 3.6	24.5 ± 3.6	31.2 ± 4#	25.3 ± 1.4*	<0.001
Ferriman score	3 (2.25-3.75)	0 (0-0.15)	12 (11-15.5)#	7.5 (2.5-10.3)*	<0.001
SBP (mmHg)	115 ± 11	121 ± 11	125 ± 3#	116 ± 9	ns
DBP (mmHg)	69 ± 7	76 ± 8	74 ± 6	72 ± 5	ns
Glucose (mg/dL)	84 ± 7	82 ± 4	85 ± 2	83 ± 6	ns
Insulin (mU/L)	6.9 ± 2.6	8.4 ± 2.9	14.1 ± 6.7	10.7 ± 6	0.02
HOMA-IR index	1.4 ± 0.6	1.7± 0.5	3.0 ± 1.4	2.3 ± 1.4	0.04
Total Chol (mg/dL)	146 ± 18	208 ± 55	158 ± 33	227 ± 11.6*	<0.001
HDL-Chol (mg/dL)	50.7 (41-65)	82.6 (61-103)	43 (39-52)	65.3 (54-78)*	<0.001
LDL-Chol (mg/dL)	79 (57-109)	100 (60-128)	92 (64-120)	120 (97-142)	0.06
Triglycerides (mg/dL)	83 ± 21	127 ± 43	111 ± 54	127 ± 63	ns

Variables are shown as mean ± SD or %. Variables with skewed distribution are shown as median (25th – 75th percentile values). P-values were calculated using one-way ANOVA, $p < 0.05$ was considered statistically significant. For Tukey post-hoc; # p<0.05 in comparison to control group and *p<0.05 in comparison to PCOS group. BMI: body mass index; SBP: systolic blood pressure; DBP: diastolic blood pressure; HOMA-IR index: Homeostatic Model Assessment-Insulin Resistance index.

Supplementary Table S2. Hormonal parameters of the study population

Parameter	**Control** (n=10)	**Control-COCs** (n=6)	**PCOS** (n=7)	**PCOS-COCs** (n=5)	**p-Value**
Testosterone (ng/mL)	0.5 ± 0.1	0.6 ± 0.1	1.0 ± 0.8	0.7 ± 0.3	ns
SHBG (nmol/L)	47 ± 14	160 ± 64#	22.6 ± 7	114 ± 84*	<0.001
FAI (%)	4 ± 1.1	1.6 ± 0.9	17.5 ± 16#	3.3 ± 2.2*	0.004
Androstenedione (ng/mL)	3.0 ± 0.9	2.6 ± 0.3	4.2 ± 1.2	3 ± 0.7	0.01
FSH (mUI/mL)	6 ± 1.4	7.6 ± 2.3	5.8 ± 2.1	5.2 ± 2.2	ns
LH (mUI/mL)	4.6 ± 1.5	4.4 ± 2.7	10.4 ± 10	5.7 ± 3.7	ns
Estradiol (pg/mL)	68.9 ± 24	75.8 ± 21	120 ± 132	74 ± 48	ns
17-OHP (ng/mL)	1.4 ± 0.4	1.4 ± 0.3	2.0 ± 1.2	1.5 ± 0.4	ns
AMH (ng/mL)	4.4 ± 2.5	3.4 ± 0.9	8.5 ± 2.6#	5.2 ± 1.4	0.001

Variables are shown as mean ± SD or %, as appropriate. P-values were calculated using one-way ANOVA, $p < 0.05$ was considered statistically significant. For Tukey post-hoc; # $p<0.05$ in comparison to control group and *$p<0.05$ in comparison to PCOS group. SHBG: sex hormone-binding globulin; FAI: free-androgen index; FSH: follicle-stimulating hormone; LH: luteinizing hormone; 17-OHP: 17 hydroxyprogesterone; AMH: anti-Müllerian hormone；ns represents not significant.
